# The importance of clinical monitoring for compliance with Continuous Positive Airway Pressure^☆^

**DOI:** 10.1016/j.bjorl.2016.05.013

**Published:** 2016-07-14

**Authors:** Lucas B. Pelosi, Mariana L.C. Silveira, Alan L. Eckeli, Emilia M.P.C. Chayamiti, Leila A. Almeida, Heidi H. Sander, Daniel S. Küpper, Fabiana C.P. Valera

**Affiliations:** a Universidade de São Paulo (USP), Faculdade de Medicina de Ribeirão Preto, Ribeirão Preto, SP, Brazil; b Universidade de São Paulo (USP), Faculdade de Medicina de Ribeirão Preto, Departamento de Oftalmologia, Otorrinolaringologia e Cirurgia de Cabeça e Pescoço, Ribeirão Preto, SP, Brazil; c Universidade de São Paulo (USP), Faculdade de Medicina de Ribeirão Preto, Divisão de Neurologia, Ribeirão Preto, SP, Brazil; d Secretaria Municipal da Saúde de Ribeirão Preto, Ribeirão Preto, SP, Brazil

**Keywords:** CPAP, OSAS, Apnea, Compliance, Positive pressure device, Obstructive sleep apnea, CPAP, SAOS, Apneia, Adesão, Aparelho de pressão positiva, Apneia obstrutiva do sono

## Abstract

**Introduction:**

Obstructive sleep apnea syndrome is currently a public health problem of great importance. When misdiagnosed or improperly treated, it can lead to serious consequences on patients’ quality of life. The gold standard treatment for cases of obstructive sleep apnea syndrome, especially in mild to severe and symptomatic cases, is continuous positive airway pressure therapy. Compliance with continuous positive airway pressure therapy is directly dependent on the active participation of the patient, which can be influenced by several factors.

**Objective:**

The objective of this study is to describe the factors related to compliance with continuous positive airway pressure therapy, and to analyze which associated factors directly influence the efficiency of the treatment.

**Methods:**

Patients who received continuous positive airway pressure therapy through the Municipal Health Department of the city of Ribeirão Preto were recruited. A structured questionnaire was administered to the patients. Compliance with continuous positive airway pressure therapy was assessed by average hours of continuous positive airway pressure therapy usage per night. Patients with good compliance (patients using continuous positive airway pressure therapy ≥4 h/night) were compared to those with poor compliance (patients using <4 h/night).

**Results:**

138 patients were analyzed: 77 (55.8%) were considered compliant while 61 (44.2%) were non-compliant. The comparison between the two groups showed that regular monitoring by a specialist considerably improved compliance with continuous positive airway pressure therapy (odds ratio, OR = 2.62).

**Conclusion:**

Compliance with continuous positive airway pressure therapy is related to educational components, which can be enhanced with continuous and individualized care to patients with obstructive sleep apnea syndrome.

## Resumo

**Introdução:**

Síndrome da apneia obstrutiva do sono (SAOS) é, atualmente, um problema de saúde pública de suma importância. Quando mal diagnosticado ou tratado inadequadamente, pode levar a sérias consequências na qualidade de vida do paciente. O tratamento padrão ouro para casos de SAOS, principalmente nos casos moderados a grave e sintomáticos, é o CPAP (*Continuous Positive Airway Pressure*). A adesão ao CPAP depende diretamente da participação ativa do paciente, que pode ser influenciada por vários fatores.

**Objetivo:**

O objetivo deste estudo é descrever os fatores relacionados à adesão ao CPAP, e analisar quais fatores associados influenciam diretamente na eficiência do tratamento.

**Método:**

Foram recrutados pacientes que receberam CPAP pela Secretaria Municipal de Saúde de Ribeirão Preto. Um questionário estruturado foi aplicado ao paciente. A adesão ao CPAP foi avaliada pela média de horas de uso do CPAP por noite. Pacientes com boa adesão (pacientes em uso de CPAP ≥4 horas/noite) foram comparados aos com má adesão (pacientes em uso <4 horas/noite).

**Resultados:**

No estudo, 138 pacientes foram analisados: 77 (55,8%) foram considerados aderentes e 61 (44,2%) foram não aderentes. A comparação entre os dois grupos demonstrou que o seguimento regular com especialista melhorou consideravelmente a adesão ao CPAP (*Odds Ratio*, OR = 2,62).

**Conclusão:**

A adesão ao CPAP está relacionada a componentes educacionais, que podem ser aprimorados com assistência contínua e individualizada ao paciente com SAOS.

## Introduction

The Obstructive Sleep Apnea Syndrome (OSAS) is now considered a major public health problem, with a prevalence, respectively, of 4% and 2% in males and females.^1^ In a recent epidemiological study conducted in the city of São Paulo, the prevalence of OSAS among adults was 32.8%.^2^ In undiagnosed or untreated cases, OSAS can lead to significant and permanent impact on the quality of life of patients, reducing overall cognitive function.^3^ In addition, OSAS is related to a high rate of morbidity and mortality, and is associated with diseases such as systemic hypertension,^4^ acute myocardial infarction and ischemic stroke.

The pathophysiology of OSAS involves the upper airways during sleep. The main associated factors are anatomic obstructive factors (such as adenotonsillar hypertrophy, base of tongue hypertrophy and neck masses), neuromuscular changes, fat distribution in the cervical region,^5^ or an association of these conditions.

The gold standard treatment for OSAS, especially in mild to severe and symptomatic cases, is CPAP (Continuous Positive Airway Pressure).^6^ CPAP is a non-invasive,^7^ easy to use and highly effective treatment. When well suited to this treatment, the compliant patient shows significant improvement in quality of life, with reduction of daytime sleepiness and^8^ reduction of cardiovascular risk.^8^, ^9^ However, this benefit does not occur if the patient does not adhere to treatment. Thus, compliance with treatment is an important negative impasse in the management of OSAS, and directly depends on the involvement of the patient.^7^

According to the literature, several characteristics can influence compliance with CPAP: other health problems in addition to OSA, the availability of polysomnography to titrate the pressure to be ideally used in CPAP and psychosocial and economic factors.^10^

A few years ago, an agreement between the Secretaria de Saúde de Ribeirão Preto and the Hospital das Clínicas da Faculdade de Medicina de Ribeirão Preto (HCFMRP) – Universidade de São Paulo optimized access to CPAP among citizens. Once the equipment had been prescribed by the specialist, the Health Department provided the CPAP to patients free of charge. Thus, the economic problem, a particularly important obstacle in developing countries, has been eliminated.^11^

The aim of this study was to observe the compliance with CPAP in those patients and to analyze the possible related factors.

## Methods

Patients who received CPAP from Secretaria Municipal de Ribeirão Preto were invited to participate in the study. They were instructed to attend the Ambulatório dos Distúrbios do Sono do HCFMRP, bringing CPAP equipment with them.

Participants who attended the invitation responded to a pre-established questionnaire addressing age, associated diseases such as depression, systemic arterial hypertension (SAH), diabetes, hypothyroidism, acute myocardial infarction (AMI), ischemic stroke (stroke), asthma and gastroesophageal reflux (GERD), smoking, drinking, and drugs in use. The historical time of diagnosis of OSAS and CPAP use (in years) was recorded, as well as whether the patient was using nasal or face mask. Finally, whether the patient was followed up in public or private health service was recorded.

In addition, patients were asked about what they knew about their disease (OSAS), through two questions: (a) “Do you know what disease you have that is treated with CPAP?”; (b) “Do you know what possible complications occur if the OSAS is not treated properly?”. The degree of satisfaction with treatment was approached by the question: “How satisfied are you with your treatment?” Daytime sleepiness was evaluated by the Epworth Sleepiness Scale (ESS).^12^, ^13^

The patients were then submitted to the general and ENT physical examination. The following components were recorded: body mass index (BMI), neck circumference, rhinoscopy (with observations of any septal deviation and characteristics of the nasal conchae) and oroscopy (with grading of tonsils, palate and tongue, respectively, by Brodsky, Mallampati and Friedman Classifications).^14^ The results of diagnostic polysomnography were also recorded, when available.

Compliance with CPAP was determined by averaging of hours of use per night, identified on the hour meter in the equipment. On the equipment that already provided the average per night, this information was recorded. On the equipment that had only the information of hours of use, the number was recorded and the patient appeared in a new return, on average one to two months after the first visit, for a new record of the hour meter and calculation of average per night. Additionally, the pressure used in the CPAP (cm/H_2_O) was recorded.

Patients were divided into two groups: Group 1 – patients with good compliance with CPAP (mean CPAP use >4 h per night); and Group 2 – those with inadequate compliance (mean CPAP use <4 h per night).^15^ After identifying the fact that the patient was not in proper use of equipment, patients in Group 2 received guidance on the importance of its optimal use, and those with no monitoring were told to return to the institution/clinic where the equipment was provided. Patients with incomplete data were excluded.

The comparison between the two groups was performed through the following tests:

– Fisher's exact test to compare frequency data such as: gender, associated diseases, type of service where he/she was being monitored (public or private), type of mask used and information on knowledge or clinical monitoring;
– Unpaired Student's *t* test was used to compare the data: age, hours of use of CPAP, Apnea and Hypopnea Index (AHI), BMI, ESS and years of diagnosis of OSA and use of CPAP.

Finally, the number of hours of use of CPAP was correlated with the parameters of BMI, age, AHI, pressure used in CPAP and ESS. For this purpose, Pearson correlation test was used. For all statistical analyzes, the differences were considered significant at *p* < 0.05.

The project was approved by the local Ethics Research Committee with number 06340012.0.0000.5440.

## Results

One hundred and eighty patients attended the consultation; of these, for 75 patients a single visit was enough to get the average of hours of CPAP use; for the other 105 patients, the second consultation was necessary to obtain this information. Of these, 42 were excluded because they did not attend a return visit. Thus, 138 patients completed all phases of the study, including 63 (45.6%) women and 75 (54.4%) men. The mean age (±standard deviation) was 58.20 ± 11.02 years and the mean BMI (±standard deviation) 33.19 ± 6.20 kg/m^2^.

AHI data was obtained from 83 patients, with the mean value being 45.52 ± 26.56. Twenty-seven of these patients had moderate OSA and comorbidities, while 56 had severe OSA. Overall, the associated diseases were present at the following frequencies: hypertension in 87 patients; DM in 39; depression in 31; hypothyroidism in 18; asthma in 13; and GERD in 19 patients. Five patients had a history of previous myocardial infarction or stroke. Thirty-seven were or had been smokers, while 13 had alcohol abuse history.

In general, the percentage of patients receiving CPAP and who had compliance considered adequate was 55.8%. Seventy-seven patients formed Group 1 (good compliance with CPAP) and 61 Group 2 (poor compliance with CPAP). The average hours of CPAP use in Group 1 was 6.13 ± 0.15 h, and in Group 2 of 1.05 ± 0.17 h. The mean difference among the groups was 5 h, which was significantly statistic (*p* < 0.0001, 95% CI 4.63–5.53).

The comparison of demographics between the two groups was not significant, either for age, BMI or AHI diagnosis (Table 1). In 83 patients who had information of AHI, the correlation between AHI and the number of hours of use was not significant (*r* = 0.18; 95% CI −0.03 to 0.38; *p* = 0.09).

**Table 1:** Comparison of demographic data between patients in Group 1 (good adherence to CPAP) and of Group 2 (poor adherence to CPAP), through Student's *t* test.

Parameter (average ± SD)	Group 1	Group 2	*p*
Age	57.84 ± 1.36	58.67 ± 1.28	0.65
BMI	32.88 ± 0.66	33.48 ± 0.89	0.59
AHI at diagnosis	47.6 ± 4.10	40.52 ± 3.92	0.21

AHI, apnea and hypopnea index; BMI, body mass index; SD, standard deviation.

There was also no significant difference between the groups for gender, presence of hypertension, DM, depression, hypothyroidism, myocardial infarction, stroke, asthma, and GERD, and the habits of smoking and alcohol consumption (Table 2). Although there was no difference in prevalence between the groups, patients with depression use significantly fewer hours of CPAP than those without depression (4.05 ± 0.27 vs. 2.24 ± 0.33; *p* < 0.0001). The difference persists being not significant for other diseases.

**Table 2:** Comparison of demographic data and frequency of associated diseases between Group 1 (patients with good adherence to CPAP) and Group 2 (patients with poor adherence to CPAP), through Fisher's exact test.

Parameter	Group 1 (%)	Group 2 (%)	*p*
Gender	54.5	45.9	NS
SH	62.3	63.9	NS
DM	29.8	26.2	NS
Depression	18.2	27.4	NS
Hypothyroidism	14.3	11.4	NS
Previous AMI/stroke	2.6	4.9	NS
Asthma	9.0	9.8	NS
GERD	9.0	9.8	NS
Smoking	24.6	29.5	NS
Alcohol abuse	10.3	8.2	NS

SH, systemic hypertension; DM, diabetes mellitus; AMI, acute miocardial infarction; GERD, gastroesophageal reflux disease.

Knowledge of the disease, its implications and possible complications were statistically different between the groups. Among patients in Group 1, 56 of 77 (72.7%) showed adequate knowledge of OSAS, while only 34 of 61 (55.7%) in Group 2 mentioned it (*p* < 0.05; 95% CI 0.99–2.03). Having adequate knowledge about the disease increased compliance with CPAP, with odds ratio (OR) of 1.42.

**Figure 1:**
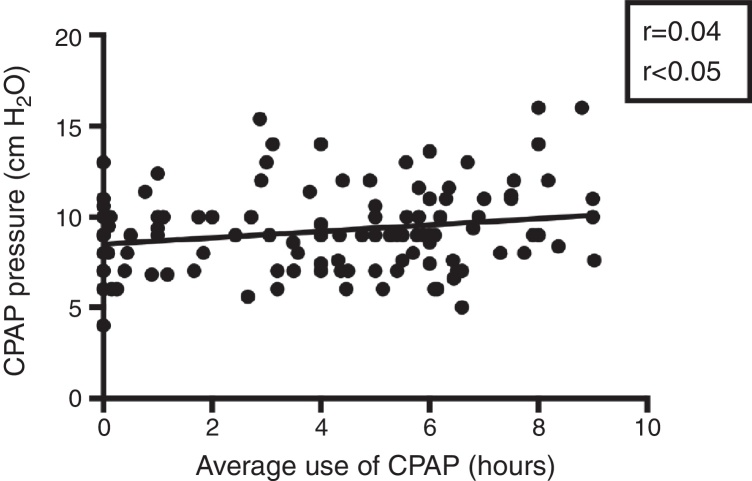
Correlation between the average hours of use of CPAP and CPAP pressure used in the patients analyzed. Study with Pearson's linear regression.

Complaints about adherence were reported by 44 of the 61 patients in Group 2 and 38 of the 77 patients in Group 1 (*p* < 0.01; 95% CI 0.49–0.89). Although CPAP medium pressure was not significantly different between groups (mean CPAP pressure of 8.95 ± 0.34 cm/H_2_O for Group 1 vs. 9.46 ± 0.27 cm/H_2_O for Group 2, *p* = 0.25; 95% CI 0.36–1.38), the number of hours of CPAP use is positively correlated to its average pressure (*r* = 0.04; *p* < 0.05), suggesting that the higher the pressure, the more compliant the patient will be with treatment (Fig. 1).

The time of diagnosis and CPAP use did not influence compliance: patients from Group 1 had a diagnosis of OSAS for a period of 3.56 ± 0.32 years, while those from Group 2 were diagnosed 3.53 ± 0.29 years (*p* = 0.94) before. Patients from group 1 received CPAP and were using it for an average of 2.90 ± 0.21 years compared to 2.91 ± 0.24 years for Group 2 (*p* = 0.99).

Regular monitoring (at least every 6 months) was reported by 64 of 77 (83.1%) patients of Group 1, compared with 26 of 61 (42.6%) in group 2 (*p* < 0.0001; 95% CI 1.62–4.25). Regular follow-up with a specialist was related to greater compliance with CPAP, with OR = 2.62. This is especially important when one considers that the groups were in CPAP treatment for the same period of time.

The compliance rate among patients who were referred by the Private Medical Service was 51.3% and in the Public Service was 60.6%. In this region, there is only one public service to follow these patients that offers therapy with multidisciplinary approach (involving otolaryngologists, neurologists, pulmonologists, physiotherapists, nutritionists and speech therapists). This could explain the better result in the Public Service. Importantly, this difference was not statistically significant.

**Figure 2:**
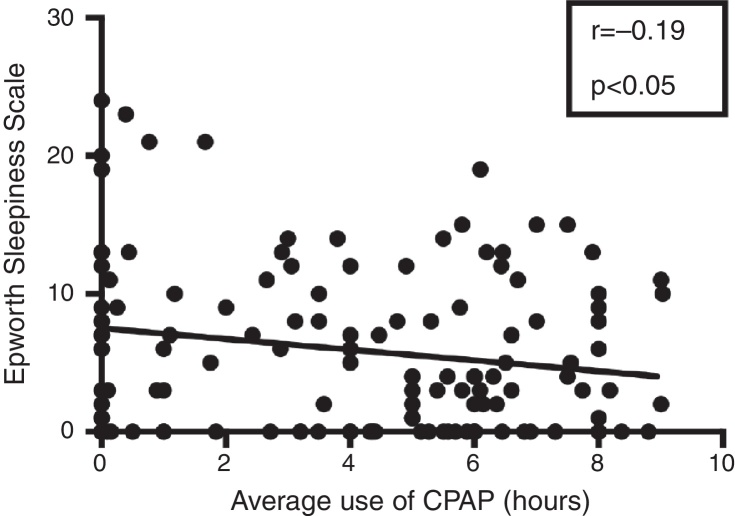
Correlation between average hours of CPAP use and Epworth Sleepiness Scale on patients analyzed. Study with Pearson's linear regression.

The average Epworth Sleepiness Scale (ESS) was significantly lower in Group 1 (4.74 ± 0.57) than in Group 2 (7.56 ± 0.86) (*p* < 0.01; 95% CI −4.86 to −0.75). A negative correlation between the average hours of CPAP use per night and ESS was observed (*r* = −0.19; *p* < 0.05) (Fig. 2). This information confirms that the more the patient uses CPAP during the night, the less he/she will present daytime sleepiness.

The type of mask was not related to compliance with CPAP (*p* = 0.161, 95% CI 0.41 to −1.18). Importantly, most patients used nasal masks (88.3% in Group 1 and 78.7% in Group 2), which greatly impaired the statistical comparison between these two groups.

Among the 138 patients, one was using BiPAP (with average usage of 0 h/night), 4 on use of auto CPAP (with use average of 0.27 ± 0.48), 54 on use of CPAP with expiratory relief (use average of 4.38 ± 0.36) and 79 in use of basic CPAP (use average of 3.78 ± 0.33). The comparison between the latter two modes of CPAP (expiratory relief and basic) was not significant (*p* = 0.22; 95% CI −1.57 to 0.36).

The ENT clinical parameters (including tonsil size, the Mallampati scale and Friedman scale) did not influence the compliance with CPAP.

## Discussion

Among the different therapeutic modalities for OSA, the positive impact of CPAP is clearly established, both for the improvement in quality of life and the positive effects on morbidity and mortality rates.^16^ Patients undergoing treatment have healthier and more productive lives and are less exposed to cardiovascular risks and accidents. However, CPAP efficiency in improving sleep architecture and reducing daytime drowsiness is strongly influenced by patient compliance.

In studies that have defined adequate compliance with the use of CPAP as at least 4 h a night, compliance rate ranged from 29% to 83%.^17^ Consequently, the main obstacles in CPAP treatment are directly related to this factor. In a recent study,^18^ it was shown that the rate of compliance with CPAP, using these same criteria of the present study, was around 65% among patients in active follow-up, a value greater than that observed in the current study.

The positive impact of CPAP in reducing sleepiness was confirmed in this study by significantly higher values in ESS in maladaptive patients. Furthermore, there was a significant inverse correlation between the hours of CPAP use by night and ESS, that is, a reduction of somnolence. These results corroborate those described by Patel et al.^19^ who, in a meta-analysis, showed that, compared to placebo, the treatment groups were able to reduce ESS, on average, 2.9 points.

In our cohort, patients with depression had lower rates of hours of use than patients without depression. This result was also suggested by Law et al.^20^ No relation with compliance rate was evident for other associated diseases. Importantly, except for systemic hypertension, other comorbidities were not common, which makes the statistical analysis less reliable.

It is well established that the diagnosis of OSAS is most frequent in obese, male and older individuals.^21^ On the other hand, it is important to identify which factors are associated with compliance with CPAP treatment, and which patient groups have higher risk of quitting therapy. This information is essential for programming specific approaches to improve the efficiency of therapy with CPAP. After evaluating 4281 patients, Woehrle et al.^22^ reported that compliance with CPAP is higher in older and male patients, and concluded that the age and gender influence the compliance with treatment, although they did not consider these data clinically relevant. Moreover, Budhiraja et al.^23^ and Queiroz et al.^18^ observed no influence of gender on treatment compliance. In this study, age and gender are not statistically correlated to the compliance rate. Regarding BMI, in contrast with what Shapiro et al. mentioned,^24^ there was no relationship of this parameter with compliance rate.

There are no studies directly correlating the compliance rate to Mallampati Scale. Zonato et al.^25^ demonstrated a positive correlation between Mallampati Scale and AHI. Queiroz et al.^18^ reported that a higher AHI at diagnosis was associated with higher compliance rate, probably because the most severe OSAS patients are more symptomatic or more concerned with the impact of OSAS. Although this association was not observed in the present study, we found a direct (but of low intensity) correlation between the CPAP pressure used by the patient, and the average number of hours of use. This correlation suggests that patients with severe obstructive factors are more compliant with CPAP treatment.

Additionally, the type of mask (nasal or facial) did not affect adherence in this cohort; this statistical analysis, however, was impaired since most of the patients used nasal masks. We also did not have any statistical difference of hours of use among patients who use basic CPAP or CPAP with Expiratory relief. These results are in line with those presented by Bakker et al.^26^ and Kushida et al.,^27^ but contradict the findings of Chihara et al.^28^

According to the results of this study, awareness of OSAS and its implications seems to play a key role in compliance with CPAP. This information is important as it stimulates the need to develop strategies to improve patients’ perception of their disease through educational programs for this population. La Piana et al.^29^ showed that educational programs significantly increase compliance with CPAP, especially during the first year. After this period, the rate tends to decrease.

This study suggests that regular and multidisciplinary monitoring after diagnosis and initiation of therapy significantly increases the chances of compliance success, probably by facilitating measures presented to patients, and because temporary problems could be more easily resolved when readily identified and properly addressed.

Considering the type of health system in which the patient was inserted (Public or Private), there was no significant difference, although it is known that there is lower compliance among patients of lower socioeconomic status.^10^ This finding may be explained by the program developed through the Agreement between HCFMRP and Secretaria Municipal de Saúde de Ribeirão Preto, which includes regular monitoring of OSAS patients in a multidisciplinary specialized setting.

This is a cross-sectional cohort that did not analyze patients either prospectively or randomly. This made the analysis of some parameters such as the use of the mask not supportable. However, important information can be obtained from this study, in particular on the importance of continuous and systematic follow-up of patients who are using CPAP.

## Conclusion

The findings of this study strongly support educational and motivational components influencing compliance with CPAP. These aspects can be achieved by individualized and multidisciplinary patient care. The importance of active and regular monitoring of CPAP users was demonstrated, and it is suggested that the interdisciplinary approach facilitates the identification of the difficulties faced by the patient. This continuous approach seems to considerably facilitate compliance with CPAP.

## Conflicts of interest

The authors declare no conflicts of interest.
